# Co‐Producing Interdisciplinary Knowledge and Action for Sustainable Water Governance: Lessons from the Development of a Water Resources Decision Support System in Pernambuco, Brazil

**DOI:** 10.1002/gch2.201800012

**Published:** 2018-10-25

**Authors:** Dave D. White, Krista L. Lawless, Enrique R. Vivoni, Giuseppe Mascaro, Robert Pahle, Ipsita Kumar, Pedro Coli, Raúl Muñoz Castillo, Fekadu Moreda, Marcelo Asfora

**Affiliations:** ^1^ Decision Center for a Desert City Arizona State University 21 E. 6th Street, Suite 126B Tempe AZ 85287‐8209 USA; ^2^ Columbia Water Center 842 S. W. Mudd, Mailcode: 4711, 500 West 120th Street New York NY 10027 USA; ^3^ Inter‐American Development Bank 1300 New York Avenue, N.W. Washington DC 20577 USA; ^4^ Research Triangle Institute P.O. Box 12194 Research Triangle Park NC 27709‐2194 USA; ^5^ Senior Hydrological Modeler Agência Pernambucana de Águas e Clima Avenida Cruz Cabugá, 1111—Santo Amaro Recife PE 50.400‐00 Brazil

**Keywords:** decision support, stakeholder engagement, sustainability, water management

## Abstract

One of the most pressing global challenges for sustainable development is freshwater management. Sustainable water governance requires interdisciplinary knowledge about environmental and social processes as well as participatory strategies that bring scientists, managers, policymakers, and other stakeholders together to cooperatively produce knowledge and solutions, promote social learning, and build enduring institutional capacity. Cooperative production of knowledge and action is designed to enhance the likelihood that the findings, models, simulations, and decision support tools developed are scientifically credible, solutions‐oriented, and relevant to management needs and stakeholders' perspectives. To explore how interdisciplinary science and sustainable water management can be co‐developed in practice, the experiences of an international collaboration are drawn on to improve local capacity to manage existing and future water resources efficiently, sustainably, and equitably in the State of Pernambuco in northeastern Brazil. Systems are developed to model and simulate rainfall, reservoir management, and flood forecasting that allow users to create, save, and compare future scenarios. A web‐enabled decision support system is also designed to integrate models to inform water management and climate adaptation. The challenges and lessons learned from this project, the transferability of this approach, and strategies for evaluating the impacts on management decisions and sustainability outcomes are discussed.

## 1 Introduction

Following the United Nations Rio+20 Summit in Brazil in 2012, the U.N. advanced the 2030 Agenda for Sustainable Development with the goal to inspire a global transition toward a sustainable and resilient planet through bold and transformative change.^1^ The foundation of the agenda is the list of 17 sustainable development goals (SDGs) and 169 targets, which were designed to build upon the successes and address the failures of the millennium development goals (MDGs).^2^ The SDG framework aims to integrate environmental, social, and economic goals and recognize tradeoffs and synergies between priorities. To achieve the goals will require mobilizing the global policy community to embrace and implement the framework and motivating the global scientific community to develop knowledge about environmental risks and strategies for enhancing resilience and sustainability.^3^ Furthermore, scientists, policymakers, and a variety of affected stakeholders at all levels and sectors of society will need to coordinate to integrate science into the societal transition process.

One of the most pressing global challenges for sustainable development in the era of the Anthropocene is freshwater management.^4^ Water is a fundamental human necessity and essential to improve social equity, promote broad economic development, and protect the functioning of the earth system. Global freshwater use has been identified as one of nine planetary boundaries regulating the safe operating space of Earth to support humanity.^5^ Freshwater is addressed specifically in SDG 6, which is to “ensure access to water and sanitation for all.” Furthermore, progress on SDG 6 is likely to have positive corollary effects upon ending poverty and hunger (SDG 1 and 2), ensuring health and well‐being (SDG 3), and promoting economic growth (SDG 8). The eight targets for SDG 6 address access, affordability, quality, sanitation and hygiene, efficiency, cooperation, and participatory decision‐making, as well as integrated water resources management. It follows that improving water governance at all levels of society is an important sustainable development objective.

Water governance encompasses a set interacting social, economic, and political systems that enable society to develop, plan, and manage freshwater resources.^6^, ^7^ Water governance includes formal and informal institutions, rules and practices, and the collective actions of public, private, and civil sector actors, and how these institutions and practices affect decisions about water resources.^8^, ^9^, ^10^ Specifically, sustainable water governance is a normative, goals‐directed framework to organize these societal activities to ensure adequate, equitable, and safe water to support economic development and social well‐being, while not jeopardizing life‐supporting ecosystems.^11^, ^12^ This process is grounded in local social, economic, cultural, and political context. Sustainable water governance requires coordination of diverse actors and their actions across the full range of water‐related activities.^12^ Sustainable water governance also requires interdisciplinary knowledge about environmental and social processes as well as participatory strategies that bring scientists, managers, policymakers, and other stakeholders together to cooperatively produce knowledge and solutions, promote social learning, and build enduring institutional capacity.^13^, ^14^, ^15^

Water governance, especially in the era of the Anthropocene, requires decision‐making under conditions of deep uncertainty about future environmental conditions, possible sociopolitical scenarios, and the evolving needs and perspectives of proximate stakeholders.^16^, ^17^ To deal with this complexity and uncertainty requires more than simply delivering scientific knowledge to the doors of decision‐makers and hoping that such knowledge is relevant and useful.^18^, ^19^ Rather, to enhance the relevance and impact of scientific knowledge requires participatory processes attuned to the needs of both scientists and decision‐makers.^20^ Furthermore, the coproduction of effective knowledge and decision support requires understanding how actors and institutions create, circulate, and use knowledge as well as social networks and power relations between actors.^21^

To address these challenges, in this research we employ a sustainability science approach that combines interdisciplinary science with stakeholder engagement.^22^, ^23^, ^24^, ^25^ This cooperative production of knowledge and action is designed to enhance the likelihood that the scientific findings, models, simulations, and decision support tools developed are scientifically credible, solutions‐oriented, and relevant to management needs and stakeholders' perspectives.^20^, ^21^ The practical goal of the project is to work with a local agency and its partners in Pernambuco, Brazil, to co‐produce knowledge and a decision support system to inform water management and climate change adaptation. The scientific goal of the project is to contribute to scholarship on the impact of interdisciplinary research on water governance through theoretically informed and experience‐based guidelines.

We present and evaluate an applied sustainability research project, in light of a set of principles and challenges for ideal‐typical transdisciplinary sustainability research, illustrated by the experiences of an international collaboration among the Inter‐American Development Bank, Arizona State University, Columbia University, Research Triangle Institute, the Pernambuco Agency for Water and Climate, and regional stakeholders in Pernambuco, Brazil. The project aims to improve local capacity to manage existing and future water resources efficiently, sustainably, and equitably. Together, the team developed systems to model and simulate rainfall, reservoir management, and flood forecasting that allow users to create, save, and compare future scenarios. We developed a web‐enabled decision support system designed to augment existing capacity and integrate multiple models to inform short‐term water management decision‐making and long‐term climate adaptation planning. The decision support system is designed to serve as a boundary object to facilitate interaction among scientists, managers, and interested stakeholders.

In this paper, we next review literature that informed the design of the project and then describe the study background. We turn to the processes and outcomes of the interdisciplinary science and summarize key findings. We close with a discussion of the challenges and lessons learned from our project, the transferability of our approach, and strategies for evaluating the impacts on management decisions and sustainability outcomes.

## 2 Inter‐ and Transdisciplinary Science and Co‐Development of Boundary Objects

Interdisciplinary science is a knowledge enterprise to integrate, interact, link, focus, and blend disciplines toward a common goal, foster mutual learning, and solve real‐world problems.^26^, ^27^, ^29^ The method emerged from applied research to integrate technological, social, and scientific knowledge.^27^, ^28^, ^29^ Interdisciplinary teams seek to develop mutual understanding of fundamental assumptions about philosophy of science underlying their work (e.g., ontology, epistemology, methodology, and axiology) to create common language.

For decades, scholars have called for integrated research approaches that transcend disciplinary silos to catalyze innovation and enhance the contribution of science and technology to address pressing societal challenges.^30^, ^31^, ^32^ A seminal cross‐national report published by the OECD Center for Education and Innovation in 1972 identified the origins of interdisciplinarity: a) within science itself as disciplines developed, fractured, and reorganized; b) in students' protest movements as students demanded that universities reorganize around pressing social problems; c) within praxis, as scientists engaged with other professionals outside the university; and d) within the needs of society, as actors outside of universities brought problems to the attention of scientists.^33^ While the OECD report argued persuasively for interdisciplinary approaches to university research and teaching, the follow‐up report 15 years later found little evidence of progress and instead noted that universities' departments had become more entrenched around conventional disciplines.^26^

Extending concepts from interdisciplinary science, transdisciplinary research developed as a way to transcend conventional natural and social science disciplinary assumptions, address real‐world challenges, and engage communities.^21^, ^34^, ^35^ Transdisciplinary science may enhance salience, credibility, and legitimacy of knowledge because multiple fields of expertise improve the likelihood that findings are accurate, applicable, and responsive to diverse perspectives.^20^, ^21^, ^34^, ^39^ In transdisciplinary work, there are numerous experts reviewing each other's work from diverse ontological, epistemological, and methodological assumptions; thus, increasing critical review. The literature notes that transdisciplinary sustainability solutions are context specific, which may be a limiting factor, and thus additional research is needed to determine the transferability of results and solutions.

Several authors have proposed systematic approaches for inter‐ and transdisciplinary sustainability science for meaningful impact.^27^, ^34^, ^36^, ^37^ These scholars argue that developing successful sustainability solutions requires participation among scientists, stakeholders, engaged citizens, and decision‐makers.^22^ Further, such authors argue that combining disciplines is urgently required to inform sustainability transitions and prevent significant degradation of human life and the earth system, especially under climate change. The demand for diverse approaches in sustainability science and research is becoming more apparent through the transdisciplinary, community‐based, interactive, and participatory approaches in the literature.^27^, ^34^, ^38^, ^39^ In response, some universities are restructuring to support transdisciplinary sustainability research.^40^

Lang et al. define the contours of transdisciplinary research to include a focus on societal problems, mutual learning among researchers from multiple academic disciplines as well as actors from outside research institutions, and focus on knowledge creation that is solutions‐oriented and transferable to scientific and social practice.^34^ They present a conceptual model of an ideal‐typical transdisciplinary research process that includes a) collaborative problem framing and building a collaborative research team, b) co‐creation of solution‐oriented and transferable knowledge through collaborative research, c) integrating and applying the co‐created knowledge. Lang et al. present a set of empirically derived design principles for each phase as well as challenges to be expected. For instance, challenges to the collaborative research phase include conflicting methodological standards, lack of integration across knowledge types and organizational structures, discontinuous participation, ambiguous results, and fear of failure leading to prepackaged solutions. The authors identify a critical need for inter‐ and transdisciplinary, solutions‐oriented sustainability science research to focus on better understanding of how the principles and challenges manifest under the different contextual conditions across various cases. Further, the literature on inter‐ and transdisciplinary approaches lacks studies that provide empirical evidence and experience‐based guidelines to evaluate and complement the conceptual and theoretical frameworks.^34^, ^41^ This paper contributes specifically to this gap by examining how the assertions and assumptions in the current academic discourse about transdisciplinary research materialize in a specific solutions‐oriented research context.

Related research addresses inter‐ and transdisciplinary research through the lens of knowledge co‐production processes and development of so‐called knowledge‐action systems, specifically within natural resource management and policy.^21^, ^42^, ^43^ Knowledge–action systems include social networks, future visions and expectations, and knowledge production dynamics surrounding policy, actions, and decision‐making for sustainability and resource management regimes.^21^ This approach supports engagement for researchers and practitioners without preconceived notions about who are the knowledge producers and who are the knowledge users.^34^ This collaborative research design allows for functional inclusion of qualitative and quantitative research, participation at multiple levels, and goal‐oriented co‐production of knowledge among various disciplines and stakeholder groups.^34^ The literature strongly supports that researchers must avoid ambiguity, be culturally sensitive, and be built upon field experience to produce conclusive and relevant results.

We also apply here insights from prior research on the cooperative development of models and decision support systems as boundary objects. Recent examples of boundary objects from the literature include models, scenarios, and maps.^20^, ^44^, ^45^ Boundary objects may be adopted and independently interpreted by multiple actors and institutions. Model‐based decision support systems are one type of boundary object that has become increasingly popular for linking environmental science and policy in coupled human–ecological systems. Our own prior research demonstrates that credibility and legitimacy in designing boundary objects can be enhanced as researchers listen to individual stakeholder concerns and make decisions collectively.^44^ Also, we have found that opportunities for privately or confidentially expressing opinions can be important, as some group settings may inhibit the sharing of controversial viewpoints.^46^ Finally, cooperative decision‐making with a regional focus can be enhanced through information technology using communal computer displays.^47^

In their design principles for transdisciplinary research in sustainability science, Lang et al. note that a key task in the initial problem framing phase is to collaboratively define research/boundary objects that link the scientific knowledge production process with the practical challenges or societal problems.^34^ They also note, however, the risks that either actors from scientific or practical spheres may dominate the process when designing the boundary object, thus leading to unbalanced problem ownership and potentially insufficient problem framing or legitimacy. Prior research, for instance, has documented tradeoffs between the priorities of scientific credibility, decision‐making relevance, and social and political legitimacy when developing boundary objects, divergent perspectives different stakeholder groups when evaluating specific boundary objects, and limited or insufficient problem framing embedded in model‐based decision support systems.^20^, ^45^, ^52^ Recently, the credibility, salience, and legitimacy framework have been critiqued for inadequately representing decision‐makers' priorities, with alternative criteria suggested including applicability, comprehensiveness, timing, and accessibility.^53^ Our research speaks to the boundary objects literature by providing an empirically and contextually informed lessons from a transdisciplinary sustainability science project seeking to combine scientific knowledge and practical components when designing a decision support system.

## 3 Study Context: Recife, Pernambuco, Brazil

**Figure 1:**
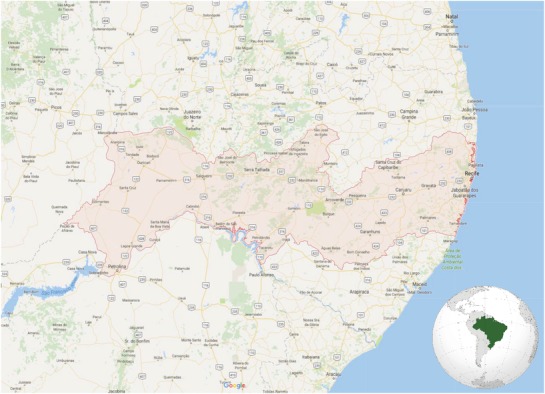
Map of study area, State of Pernambuco, Brazil. Image from Google Earth.

The setting for our project is the State of Pernambuco in northeastern Brazil, which covers 98 312 km^2^ and has a population of ≈9.2 million (**Figure** **1**). Recife is the largest city and capital and is located at the confluence of the Beberibe and Capibaribe Rivers on the Atlantic coast. Recife is home to nearly 1.6 million people and the larger Metropolitan Region of Recife (RMR) has a population of nearly 4 million. Recife is a major port, industrial center, commercial hub, and popular tourist destination.

The low average water availability, high seasonal variability, and heterogeneous climate conditions across the state contribute to significant water management challenges. Pernambuco's land area is classified as hot semiarid climate (BSh, 61%) and tropical Savanna climate (As, 33%).^48^ Brazilian National Water Agency (ANA) data show that Pernambuco has water reserves of ≈1300 m^3^ per person per year, below the 1700 m^3^ per year typically associated with water stress.^45^ The hydrological regime of the state is highly variable. The rivers of the Coastal Zone and Zona de Mata (Atlantic Forest Zone) are perennial but the rivers of the Sertão (drought zone) and Agreste (transition to drought) are intermittent. Droughts associated with El Niño conditions are common in the Sertão. Northeast Brazil has been labeled a socio‐climatic hotspot due to projected significant and adverse physical impacts and social vulnerabilities associated with climate change.^49^, ^50^, ^51^

The Pernambuco water control system is comprised of a series dams and canals along the São Francisco and Atlantic draining rivers. Reservoirs are located within 29 planning units composed of 7 internal watersheds that drain to São Francisco river, 9 groups of inland rivers (eight of which drain to São Francisco), 6 groups of coastal rivers, 5 coastal watersheds, 1 internal watershed that passes through another state before reaches the ocean, and the archipelago of Fernando de Noronha. The largest part of the Pirapama system is in Cabo de Santo Agostinho, operated by Compesa (the regional water utility company), and was completed in 2011. The system includes the Pirapama Dam with a capacity of 61 million cubic meters, the adjacent Pirapama Water Treatment Plant with a capacity of 5.13 m^3^ s^−1^, and a constellation of smaller reservoirs.

## 4 Research Approach

We modeled our research approach on the conceptual, ideal‐typical research process presented by Lang et al., which includes a) collaborative problem framing and building a collaborative research team, b) co‐creation of solution‐oriented and transferable knowledge through collaborative research, c) integrating and applying the co‐created knowledge.^34^ Our team includes relevant expertise, drawn from universities, a consulting research firm, the regional policy and management agency, and an international development funding agency. Together the team developed a joint understanding of the water sustainability challenges and an agreed upon research framework. To inform our understanding of the sustainability challenges and potential solutions, we reviewed relevant policy documents and consulted local stakeholders through multiple scoping meetings. We also conducted a survey questionnaire, focus group meetings, and key informant interviews.

The initial scoping meetings between all partners, a part of the collaborative problem framing process, identified several key water sustainability challenges that could be addressed through interdisciplinary science. These included vulnerabilities to climate change risks and extreme events (i.e., droughts and floods), uncoordinated reservoir management, the need to improve economic optimization in water allocations, and additional capacity to integrate, visualize, and communicate data. To address these challenges, we assembled an interdisciplinary team incorporating climatology, hydrology, computer science, policy analysis, visualization, and decision science. To co‐create the solution‐oriented knowledge, the team worked in consultation with implementation agents to develop and integrate models of hydroclimatology, water resource management, reservoir optimization and forecasting, and flood forecasting. To enhance the integration and application of the interdisciplinary science, we developed the decision support system with the local agency including transfer of software, open‐source code, and training materials.

## 5 Regional Water Governance System Analysis

One component of our interdisciplinary research was a systematic regional water governance system analysis.^12^, ^52^, ^53^, ^54^ This included review of key policy documents as well as an empirical assessment of stakeholders' perspectives on the sustainability of the system, based on participant observations, individual interviews, focus groups, and a survey questionnaire. Regarding the research framework, the regional water governance analysis is primarily designed to contribute to the collaborative problem framing tasks in phase A. That is, we designed these activities to help identify societal‐relevant research questions, frame sustainability problems and potential solutions from multiple perspectives, and shape and settle upon the broad design for the joint research/boundary object. Next, we examine national and regional water policy and present empirical findings.

### 5.1 Legal and Institutional Context for Water Governance in Pernambuco

The historical development of modern Brazilian water policy has been organized into three phases with distinct institutional, legal, and political contexts.^55^ In 1916, The Civil Code of Brazil marked the beginning of the “Navigability Phase.” This period lasted until the 1930s and defined rivers as communal public property. The weak regulation of this era, and its overemphasis on navigation and agriculture, lead to calls for social, legal, and political reforms during the early and middle 1930s. The reforms of the 1930s facilitated the transition to the “Hydroelectricity Phase.” This era was marked by the Water Code of 1934, which revoked the Civil Code of 1916 and classified water resources into three types of waters: public, common, and private. The new water categorizations focused on using public authorities to control the water resources. The Hydroelectricity Phase was characterized by the landmark expansion of hydroelectric power generation in the late 1930s until the 1980s when, yet again, there was a call for reforms. The last reforms helped to transition the usage of Brazilian water into its current phase which focuses on the environment. In 1981, The “Environmental Phase” of Brazilian water policy was ushered in by the National Environmental Policy Act, which acknowledged water's environmental value.

Prior to 1968, water supply and sanitation responsibilities fell upon each state's municipality. During this time, there was not an institutional structure in place to plan and finance water services. In 1968, the National Water Supply and Sanitation Plan (PLANASA) was created, which was the federal government's initiative to manage water and water sanitation throughout Brazil. In the early 1970s, the State Water and Sanitation Companies (CESBs) were established in each state to help expand water and sanitation services. The Pernambuco Water and Sanitation Company (Compesa) was established in 1971. Compesa managed the water system for two decades with relatively few changes in regulation, law, or policy. The National Water Resources Policy (NWRP) and the National Water Resource Management System (NWRMS) were enacted in 1997 with the Law 9433 for Pernambuco. The law gave value to water by defining it as a scarce resource that has multiple uses and created water agencies and state watershed committees and has helped to decentralize the management of water resources in Pernambuco.^56^ Within Pernambuco, institutional capacity for water resources management was further strengthened through State Water Resources Policy in 2005 (Law No 12984) and the Integrated Water Resources Management (SIGRH). Law No. 13205 of 2007 established the State Department of Water Resources. The Pernambuco Water and Climate Agency (APAC) was created in 2010 under State Law No. 14028 to strengthen and establish the State Policy on Water Resources.

### 5.2 Stakeholders' Assessment of Sustainable Water Governance in Pernambuco

Our empirical assessment focused on stakeholders' evaluations of APAC and its partners in sustainable regional water governance. We used a mixed‐methods' case study design to evaluate stakeholders' perceptions of sustainable water governance with data collected from survey questionnaire (*N* = 96), focus groups and interviews (*N* = 34), participant observations, and document analysis.^57^ The results revealed consensus among the respondents in their support of a set of sustainability principles derived from the literature to guide water governance decisions: i) social‐ecological system integrity, ii) precaution and adaptability, iii) social–ecological civility and democratic governance, iv) interconnectivity from local to regional to global scales, and v) intergenerational and intragenerational equity.^11^, ^54^, ^58^ Respondents rated each of the sustainability principles as very important to extremely important, with means from 4.50 to 4.71 on the five‐point scale.

We also identified significant gaps between respondents' ratings of the importance of the guiding sustainability principles and their satisfaction with the current performance of the regional water governance system on those same principles using an importance‐satisfaction analysis. Respondents were consistently unsatisfied to very unsatisfied with the status of the Pernambuco water system on those sustainability principles, with means ranging from 2.41 to 2.50. For each of the five sustainability principles, the importance of the principle was rated more highly than the satisfaction. Notably, respondents identified a significant gap between the importance of social–ecological system integrity and the ability of the current regional governance system to achieve this principle. Specific areas of concern included maintenance of minimum water flows and quality, conservation of groundwater, and coordination among resource managers and planners.

**Figure 2:**
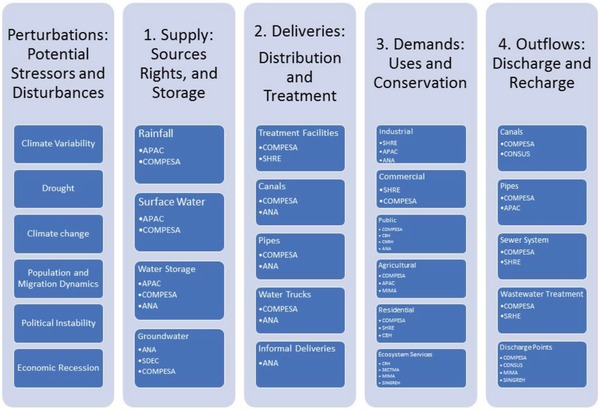
Water governance analysis diagram for Pernambuco, Brazil, identifying specific actions and actors relevant each domain of the water system.

Our results also showed that stakeholders were relatively more satisfied with the governance systems on two sustainability principles: i) precaution and adaptability and ii) social–ecological civility and democratic governance. For a sustainability transition to take place in the future, it will depend largely on effective collaboration and coordination within a participatory and multilevel (federal, state, and water shed) water governance system in Pernambuco. Multiple sources of evidence (focus groups, interviews, and survey responses) confirmed that respondents feel participatory cooperative governance organizations are genuinely important to implement reforms, looking especially to the national and state watershed committees as leaders to facilitate and include public participation in their decision‐making surrounding reform options. These same respondents, however, also identified federal and state government agencies such as the National Water Agency and APAC as influential and required. To these respondents, the key feature to the structure of sustainable water governance is collaboration and communication among various levels of decision‐makers and water managers, as opposed to centralization or decentralization of power. **Figure** **2** illustrates the key actors and actions in the Pernambuco water system. The results of the regional water governance analysis highlighted areas of convergent priorities between scientific and practitioner actors—including the potential utility of hydrometeorological modeling, flood forecasting, and reservoir management—that informed the design and development of the scientific modeling activities and the resulting boundary object.

## 6 Weather, Climate, and Water Modeling

The second component of our research included an interconnected suite of weather, climate, and water modeling activities. These scientific activities aimed to enhance water governance by improving the capacity of the regional water management agency. Team members from Arizona State University (ASU), Columbia, Research Triangle Institute (RTI), and APAC collaborated on real‐time hydrometeorological forecasting, rainfall forecasting, and reservoir optimization modeling for water management. These modeling components are then integrated through the decision support system and the capabilities transferred to APAC. The weather, climate, and water modeling activities constituted the bulk of the interdisciplinary research in the project (i.e., phase B) and thus key project‐level tasks included defining the roles, responsibilities, and accountabilities for the scientific and practitioner actors, discussing and balancing scientific rigor with societal relevance and practitioner capabilities, and establishing processes for resolving conflicts.

### 6.1 Real‐Time Hydrometeorological Forecasting

**Figure 3:**
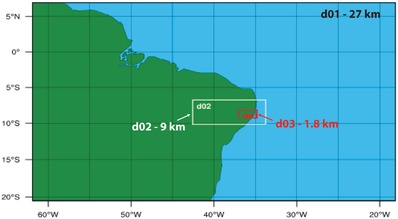
Nested domains used for the configuration of WRF and “on‐line” WRF‐Hydro. Domain 1 (d01, 27 km resolution) is the outer domain receiving the lateral boundary conditions from the general circulation model. Domain 2 (d02, 9 km) is the domain including the state of Pernambuco used by APAC to issue weather forecasts. Domain 3 (d03, 1.8 km) is the domain including the Una basin where the prototype application of WRF‐Hydro has been conducted.

One of the modeling activities was aimed at improving the real‐time hydrometeorological forecasting capabilities of APAC. Currently, the agency issues meteorological forecasts for the next 3 days using the Weather Research and Forecasting (WRF) numerical weather prediction model.^59^ These are predictions of atmospheric variables, such as precipitation and temperature, covering the entire state of Pernambuco at 9 km spatial and 6 h temporal resolution (**Figure** **3**, domain 2). A recent development of WRF includes the WRF Hydrological Modeling Extension Package (WRF‐Hydro), which allows simulating: i) the coupled atmospheric‐land surface hydrologic processes; ii) the lateral movement of water in the surface and subsurface portions of the Earth's land mass; and iii) streamflow routing in the channel network.^60^ As such, WRF‐Hydro can be utilized to issue operational streamflow forecasts at distributed locations within the channel network directly from atmospheric predictions. This capability is crucial for an agency like APAC, for anticipating storm and flooding events, issuing flood warnings or alerts, and supporting civil protection.

In this project, the research team and the hydrometeorological forecasting division at APAC conducted a prototype study to test the feasibility of the application of WRF‐Hydro in the state of Pernambuco. The study was focused on a selected watershed—the Una basin—located in the southwestern portion of the state of Pernambuco (Figure 3, domain 3). This watershed was chosen by APAC because of the frequent occurrence of intense floods that have caused significant losses in terms of property and casualties in the past.

**Figure 4:**
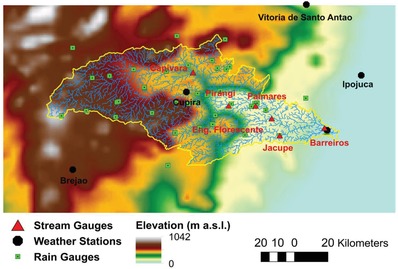
Topography and stream network of the Una basin along with location of the ground stations used to create the gridded forcings for the calibration of the land‐surface and routing schemes of WRF‐Hydro.

The first activity was aimed at calibrating the WRF‐Hydro land‐surface and routing schemes thorough uncoupled or “off‐line” simulations. These are model runs carried out with observed meteorological forcings (i.e., without any input provided by the WRF atmospheric model). Precipitation, streamflow, and meteorological data at daily and hourly resolution from ground stations (**Figure** **4**) were first collected and quality controlled. Interpolation and downscaling routines were then applied to generate the gridded datasets required by the model. The year 2012 was selected as calibration period. Following previous applications of WRF‐Hydro, a limited set of model parameters was varied to obtain the best match between observed and simulated streamflow at six nested locations in the channel network.^61^, ^62^ This was achieved in two phases. The optimal parameters controlling the transformation of rainfall into runoff were identified by evaluating model performances at the monthly scale, without activating the hydraulic routing option. Next, the values of parameters affecting the shape of the simulated hydrographs were determined through comparison with observed data at daily resolution over a period of 3 months, by applying both the subsurface and surface routing schemes.

The second activity aimed to validate the flood forecasting skill of WRF‐Hydro in a pseudo‐operational setting through coupled or “on‐line” simulations on a selected number of flood events, including a recent devastating flood in 2017. In these model runs, the WRF atmospheric model was directly coupled to the land‐surface and routing schemes. For each flood episode, the lateral boundary conditions for WRF used by APAC for their operational meteorological forecasts were used to drive the calibrated WRF‐Hydro system, which produced streamflow predictions at each location of the channel network. The peak discharge estimated by local authorities for each flood event was then compared with the value simulated by the model.

### 6.2 Rainfall Forecasting and Reservoir Optimization Modeling

To achieve optimal management of water in the state of Pernambuco, the project team developed rainfall forecasts for incorporation into the HydroBID water resources management model (see the following section). HydroBID uses the rainfall forecast inputs to provide streamflow forecasts in the reservoir optimization model. Rainfall forecasts were made using nonhomogeneous hidden Markov model (NHMM) to provide daily rainfall at a station level. For statistical downscaling, NHMM has been widely used for rainfall.^63^, ^64^, ^65^ The NHMM uses hidden states (a set of rainfall probabilities, which shows the amount of rainfall in each station) to make daily rainfall predictions.^66^ To conduct NHMM, a set of predictors, which could include different climate indices, are used to facilitate the rainfall projections. For the case of Pernambuco, the NHMM uses 90 stations across the state and forecasts are made for the period of 1 December to 31 August. These 90 stations had more than 75% data over the period selected with historical rainfall starting December 1962 to August 2016 (without the months of September, October, and November).

For the selection of predictors, correlation analyses were conducted between the December to August average rainfall period with 14 predictors using different months, two monthly and seasonal averages. These predictors included various sea surface temperature indices from the Atlantic and Pacific, wind patterns, temperature, etc. From these predictors, two had the highest correlation for May–June–July (MJJ) averages. Those two were the Niño 3.4 Index and the Tropical Atlantic Variability (TAV). The impacts of El Niño on rainfall in northeast Brazil has been documented showing a reduction in rainfall.^64^, ^67^, ^68^ The second, the TAV, is a characteristic of the strength of the interhemispheric gradient in determining the sea surface temperature (SST).^69^ This is the difference between the average SST in the tropical North Atlantic (the average between 5N and 25N, 60W and 30W) and in the tropical South Atlantic (the average between 25S and 5S, 30W and 0E). The dynamics determining the SST in the Northern and the Southern Atlantic is different, for which reason the tropical belt from 5S to 5N was dropped.^70^ The Niño 3.4 Index and the Atlantic SST data were obtained from Koningklijk Nederlands Meterologisch Instituut (KNMI) Climate Explorer (http://climexp.knmi.nl/).

For the study, with the 90 stations selected, the December‐to‐August daily data (274 days), and the two predictors (Nino 3.4 and TAV), NHMM runs were conducted using the NHMM R Package. For validation, 11 years of historical data were used, with each forecast making 100 ensemble forecasts for each year. These results are then used in HydroBID to produce streamflow forecasts for the reservoir optimization model. The reservoir optimization model uses a linear programming model to minimize the cost of meeting water demands of multiple cities from multiple cities and for a multireservoir system under climate uncertainty. The objective of the model is to minimize the expected cost of meeting these demands over the ensemble forecast (which were developed by RTI using HydroBID), through an optimal set of monthly diversions from each reservoir to its candidate demand points. Water imports from outside the system, as well as penalties for failure to meet supply targets, are considered with appropriate costs or economic penalties. The release policy can be updated on a monthly or seasonal basis using updated reservoir storage and seasonal to interannual streamflow forecast information. The novelty of the model is that it takes ensemble forecasts, which can be created through the means of streamflow forecasts. The cost of failure is also included in the study to show how much water is not supplied through reservoirs and import sources and is kept significantly high so that failure is the least desirable option.

The model is applied for the Jucazinho reservoir system in the state of Pernambuco, with 5 reservoirs, 5 water trucks, and with or without Rio São Francisco providing water to 19 municipalities. The Jucazinho system includes the reservoirs Jucazinho, Eng. Gercino Pontes, and Machado from the Capibaribe River, and Prata from Una River. Since the Rio São Francisco transfer is yet to be completed, we assessed the value of current infrastructure with that of the future. The results demonstrate that the cost of water supply can be significantly reduced when using streamflow forecast data as compared to a policy design process, which does not use any forecasts.

### 6.3 Water Resource Planning and Management Modeling

As part of its commitment to increase water resource management capacities among member countries, the Inter‐American Development Bank (IDB) sponsored the development and application of a suite of watershed modeling tools collectively known as the HydroBID modeling system. The HydroBID modeling system, built on the framework of the Watershed Flow and ALLocation model (WaterFALL), includes hydrology and climate analysis modules to estimate the availability of unimpaired freshwater at the regional, basin, and sub‐basin scales.^71^ It has two major components: Hydrologic Model and the Analytical Hydrographic Database (AHD). The hydrology model, user interface, and results viewer exist in a packaged Java class file format. The AHD, which is stored in a self‐contained, public domain SQL database engine known as SQLite, can be accessed from the open source mapping software QGIS.

The computational engine of the hydrologic model is an enhanced version of the Generalized Watershed Loading Function (GWLF).^72^ The enhanced GWLF is coupled with a novel lag‐routing methodology to compute results on a submonthly time step and to model large watersheds. A preprocessor, referred to as the Climate Data Interpolation Tool (CDIT), automates interpolation of daily temperature and precipitation time series between stations. Model output is generated as a time series of predicted streamflow, at either a daily or monthly time step. The system has a graphical user interface to facilitate loading and processing of model input, as well as to display both graphical and tabulated model output. Model parameters and river network are extracted from the AHD. The model has options incorporate sediment and reservoir routing and can be coupled with an existing MODFLOW groundwater model.

**Figure 5:**
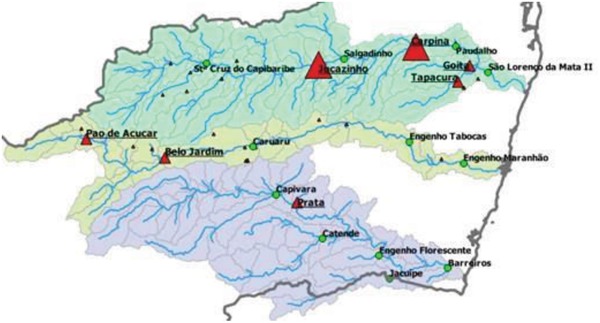
Capibaribe (blue), Ipojuca (yellow), and Una (purple) basins. Flow gauges represented by green circles with bold labels. Reservoirs represented by red triangles with underlined labels. Minor reservoirs (less than 30 million m^3^ in capacity) represented by small red triangles without labels.

For this project, HydroBID was used in Pernambuco to model daily water availability in the Capibaribe, Ipojuca, Una, and Pajeu River basins (**Figure** **5**). APAC provided local historical climate and flow observations used for model inputs. Major reservoirs in the basin were implemented using a simplified simulation model within HydroBID. Once HydroBID was calibrated at each stream gauge, the results were validated at the outlet of each basin. The verified model was extended to evaluate changes in flow regime due to precipitation and temperature change under future climate scenarios. To complement the long‐term forecasting efforts in the basin, precipitation forecasts provided by Columbia University were integrated with HydroBID to estimate a range of potential future flow scenarios.

## 7 Decision Support System

**Figure 6:**
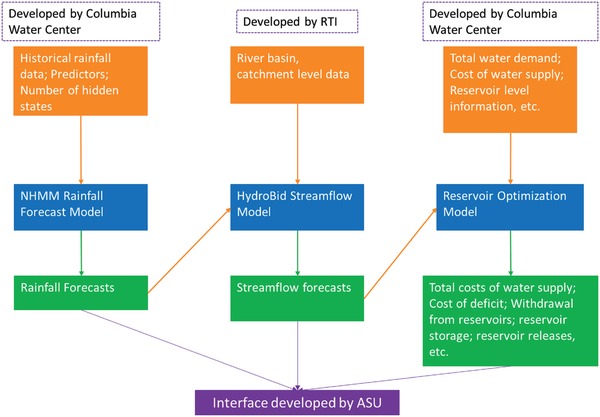
Model integration framework. Orange boxes and arrows represent inputs into the models. Blue boxes represent the models. Green boxes and arrows represent the outputs from each model. The green boxes show two arrows as the output from one model go in as input into the other model. All outputs in green are put in an interface developed by Arizona State University.

The decision support system served as the research/boundary object to structure the interactions between the interdisciplinary research teams and the management agency (i.e., research phases B and C). The knowledge production and modeling efforts described earlier are combined into a flexible online platform. Since there are two separate flows of computation, the decision support system is designed with two distinct functions (**Figure** **6**).

The first is a scenario planning tool to inform freshwater management and delivery. With the system, decision‐makers generate scenarios varying the availability of water supplies and optimizing distribution into the demand centers. In a first step, the users specify parameters for three models that run in a connected chain via a dashboard interface. The first component retrieves updated rainfall predictors from the internet and prepares them for the rainfall forecast model described earlier. Then, the rainfall forecast model computes an ensemble of 100 scenarios for 2 years into the future. The next component uses station gauge data produced by the rainfall model to estimate the amount of rainfall in each catchment for the area of interest. Next HydroBID estimates the inflow to reservoirs of interest for all 100 ensemble members. Finally, the reservoir optimization model uses the 100 inflow ensemble forecasts to optimize water allocations for the demands connected to the reservoirs with the goal of minimizing cost. The combination of input data and results is stored as a scenario that can be saved, shared, and evaluated by researchers, managers, and other stakeholders. The decision‐makers can generate their own scenarios and conduct pairwise comparisons and evaluations. They can explore viable solutions to water shortages in the area and export the result datasets and reports that can be used in presentation and for public outreach. This web‐based tool can be used jointly for several users at the same time which allows for a high degree of flexibility.

The second process is a flood monitoring tool. This functionality uses data generated by WRF‐Hydro to produce a monitoring dashboard that is displayed in the APAC command center. The WRF‐Hydro model produces streamflow estimates every 6 hours and these results are forwarded to the monitoring workflow within the decision support system (DSS) which extracts the flows at locations specified by APAC to project future flows at those locations in a threshold line chart—one chart for each location. In addition, the locations are marked on a map, so that the results are easier to interpret. In case of a flow that exceeds the specified threshold, the line above the threshold is marked red, a warning is shown and the location inside the map is marked with red. Furthermore, e‐mails can be sent to key system operators alerting them to results of the simulations when these exceed predetermined thresholds. Managers can then monitor on‐the‐ground conditions to determine what actions need to be taken.

Both functions are implemented using the ChainBuilder integration framework, which allows for adjusting, managing, processing, and visualizing the decision support system at a later point in time. All computations are executed at the Agência Estadual de Tecnologia da Informação (ATI) using a cluster (WRF‐Hydro) and several commissioned servers. The servers are secured by ATI to the internet but can be directly accessed from the APAC facility. This arrangement allows APAC to maintain full control over the servers and their models. In both cases, several submodels were integrated.

**Figure 7:**
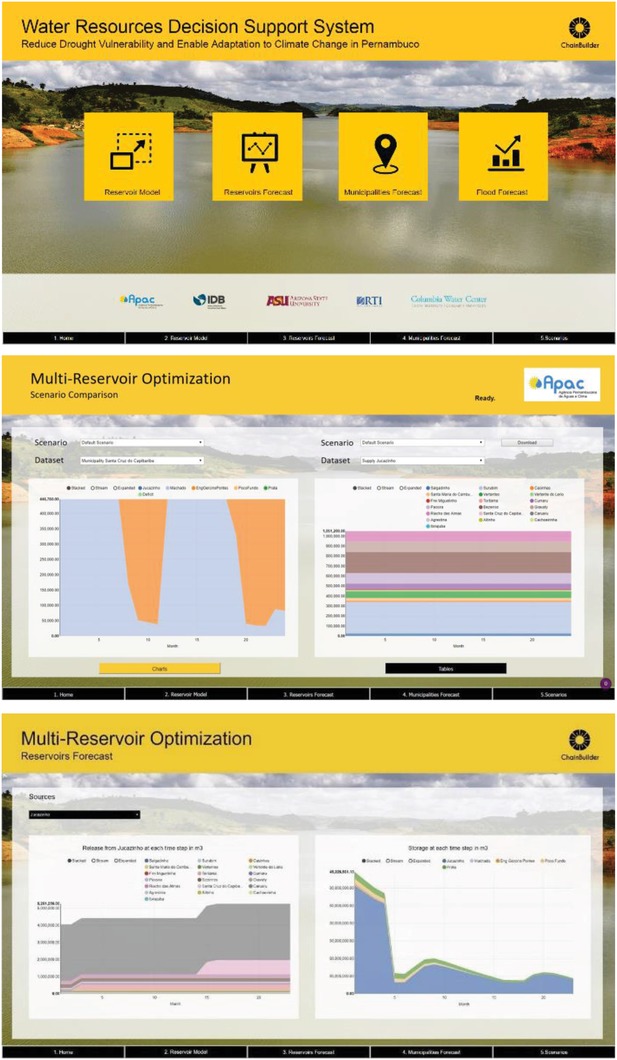
Sample dashboards for web‐based water resources decision support system.

The visualization was developed using an iterative process between the decision environment team and APAC—the final user of the products (**Figure** **7**). During several iterations, proposed display layouts were presented, criticized by the APAC, and revised to make sure that the displayed data are relevant to APAC's needs. In addition, we developed a report generator and data download component, so that the APAC team can extract relevant information for presentations or further analysis.

## 8 Discussion

Freshwater management is one of the most significant global challenges facing humanity in the Anthropocene. For science to contribute effectively to sustainable water governance, scientific knowledge and products must credible, salient, and legitimate. One approach to creating science useful for decision‐making is to follow a systematic process to build an interdisciplinary team along with stakeholders, collaboratively frame the problems, cooperatively create solutions‐focused knowledge, and to integrate and apply the knowledge and tools. Our experiences in this project illustrate the trials and tribulations of an interdisciplinary scientific team committed to producing use‐inspired knowledge. Next, we evaluate the accomplishments and potential impacts of this project in relation to the principles and conceptual framework for transdisciplinary sustainability research discussed earlier and identify where the current project lived up those principles and where it failed to do so.

### 8.1 Collaborative Problem Framing and Building a Collaborative Research Team

Key tasks for the initial project phase are to build a collaborative research team, create joint understanding of the problem, collaboratively define the research objectives and boundary object, and design a method for collaborative knowledge production.^34^ The key challenges for this phase identified in the literature include lack of problem awareness or insufficient problem framing, unbalance problem ownership, and insufficient legitimacy of the team or actors involved. Specific strategies to overcome these challenges include conducting pilot studies to build problem awareness, joint leadership, and stakeholder's mapping to create structures that enable participation.

In this project, the team established a relatively effective network with appropriate representation of multiple scientific disciplines and practitioner perspectives. The funding agency played an influential role in creating the team and serving as a liaison between the US‐based universities and the Brazilian partners. To create a functioning collaboration, we had to address common barriers to collaboration such as different languages, geographic distance, and differing organizational cultures between the funding agency, universities, research consulting firm, and local management agency. We also faced turnover in project management personnel at the funding agency as well as social, political, and economic disruptions in Brazil during the project, which affected the agency's ability to assign staff and allocate scare resources to the effort.

The governance analysis component was particularly informative for the collaborative problem framing phase. The scoping meetings, interviews, and focus groups helped us to understand the perspectives of the interacting stakeholders, especially the meetings conducted in Recife. These activities also fostered social learning and social capital between the research team and the local management agency; the policy history and stakeholder's assessments helped us to identify key issues that could be addressed through interdisciplinary science. Although we employed stakeholder mapping and stakeholder engagement exercises to enable participation, one limitation of our project was that government and scientific actors tended to dominate the research process while other interests including environmental nongovernmental organizations (NGOs), agricultural interests, and marginalized populations were underrepresented. This raises concerns about the possibility of insufficient problem framing and insufficient legitimacy of the team or actors involved. For future research, we recommend that interviewees and survey participants are more broadly representative of a fuller range of views on water management. Indeed, our initial project design included a socio‐cultural anthropologist with expertise in Brazilian water politics and plans to conduct in‐depth ethnographic research with a wider range of affected stakeholders including marginalized communities. This effort, however, could not be supported due to budget and time constraints, and this weakened the overall result. Furthermore, although we established effective consultation, social learning, and collaboration with the local university on certain project tasks, especially the hydrological modeling, our project would have been stronger with deeper and more sustained coordination with local political and social scientists. We recommend that other teams engaging in collaborative research design allocate more time and financial resources to support local university partners to overcome this limitation.

Defining the boundary object, in our case, the model‐based decision support system required sustained engagement, deliberation, and negotiation between the partners. However, the boundary objects' literature stresses their utility for structuring relations among scientists and between scientists and other stakeholders and boundary objects literature effectiveness for co‐producing knowledge in science‐policy contexts, creating these products can create tensions.^20^, ^44^ For instance, the management agency leadership, seeking to maximize the impact of the available funding and technical assistance opportunity, and taking an expansive view of the challenges and potential solutions (i.e., wide problem framing), advocated for a system that incorporated an broad view of sustainability and wide range of data and models and inform operational decision‐making, long‐term planning, and communication to policymakers. The university researchers had to balance these demands and expectations against technical capabilities and funding.

### 8.2 Co‐Creation of Solution‐Oriented and Transferable Knowledge

Key tasks for the second phase are to assign and support roles for practitioners and researchers and to apply and adjust inter‐ and transdisciplinary methods and settings to generate and integrate knowledge. Key challenges in this phase from the literature include conflicting methodological standards, lack of integration, discontinuous participation, vagueness and ambiguity of results, and fear of failure.^34^ In this project, we faced most of these obstacles and employed several strategies, with varying success, to overcome them.

Early in the project development stage, the partners held multiple scoping meetings, facilitated by the sponsor, to specify the roles for each participant. In these meetings, we negotiated the tasks and roles for each actor and organization; often the discussions were animated, as partners sometimes disagreed about the appropriate research question, method, or model to use while the sponsor managed sensitive discussions about apportioning the available funding. This process required an initial investment of time, travel, and finances from the partners, including university professors and consulting researchers who typically compete for the same grants and contracts, prior to any formal agreement, but the outcome of the discussion was a set of integrated contracts and tasks that detailed specific roles, responsibilities, and timelines with the overall project success in mind. During project implementation, however, the team creating the decision support system was forced to function as the de facto project integration managers. Since this authority was not clearly established at the outset, however, this team had to work with all partners to understand how the data, models, and partners were interacting to meet the end results for the decision environment.

The hydrometeorological modeling activities were most relevant in the co‐creation phase as the research team developed methods suitable to generate solutions for the identified sustainability problems. Here, we faced the challenge of conflicting methodological standards and conflict between scientists about the most appropriate tools. Ultimately, the team settled upon a mix of cutting‐edge research models (i.e., WRF‐Hydro) and practice‐oriented management models (i.e., HydroBID), drawing from the relevant experience of the team members and matching the tool to the task. The modeling efforts also opened collaboration between the researchers, the management agency, and local university researchers, who provided data, technical assistance, and local experience necessary to setup, calibrate, and validate the models.

### 8.3 Integrating and Applying the Co‐Created Knowledge

The essential tasks for the final phase of interdisciplinary sustainability research include generating targeted scientific and practitioner products as well as evaluating scientific and societal impact (i.e., 2D integration).^34^ Specific challenges in this phase from the literature include limited, case‐specific solution options, lack of legitimacy of transdisciplinary outcomes (i.e., friction between scientific and political processes), and distorted research results, and difficulty tracking scientific and societal impacts.

It is necessary to discuss and design these products in the early design phase to counteract the opposing pressures and accountabilities for the science and policy actors.^20^ In this effort, the team included university researchers at various stages of advancement, including graduate student, postdoctoral researcher, research scientist, assistant professor, and professor. Academic products are produced and valued differently for these career stages (i.e., thesis, single‐author publication, group publication, management report, and model version), and it is necessary to address each participant's interests. Additionally, our team included nonprofit institute researchers and management practitioners, each with specific interests and incentives to consider.

The governance analysis and decision support system efforts were helpful for the integration and application. For example, the governance analysis helps to identify specific actors and institutions that are trusted and will be instrumental to implementing sustainability transition strategies. In developing the decision system, we faced technical challenges pulling together the correct data between the components and dealing with speed of execution. The data exchange problems were solved using tight pairwise collaboration between the developer of the component and the decision environment team. Some components were redeveloped to make use of parallel execution so that the expected run times of the model workflow are minimized.

Finally, we address the question of scientific and societal impact. Scientific impact will be addressed through conventional measures including the number of researchers trained, journal articles published, and citation analyses although these metrics are inadequate for judging the contribution to sustainability outcomes.^22^ In addition to traditional performance metrics and feedback from the funding agency, a project such as this focused on cooperative production of knowledge and action should also consider other metrics, such as increased capacity in social networks and relevance and impact of scientific knowledge. These impacts are difficult to measure due to significant time lags and the complexity of assigning causality. In related research, for instance, societal impacts of transdisciplinary sustainability research were difficult to identify at the end of the project using conventional measures but were revealed in a comprehensive ex‐post evaluation 3 years later.^73^ Combining outcome‐based and social capital evaluation yields a superior measure of the value of knowledge created and the social process by which the knowledge is produced. Similarly, the ultimate impact on sustainability outcomes in Pernambuco from this project will be addressed through process‐tracing techniques and a comprehensive ex‐post evaluation with local stakeholders 1–3 years following project completion.

## 9 Conclusion

In this paper, we have presented and evaluated a sustainability research project considering a set of principles and challenges for ideal‐typical transdisciplinary sustainability research. This paper responds to calls in the literature for greater emphasis on understanding how inter‐ and transdisciplinary research plays out in specific social, political, and environmental contexts in multiple cases and provides evidence to evaluate the utility of the design principles.^34^ We conclude that, overall, this ideal‐typical transdisciplinary sustainability science approach—working with stakeholders to understand problem framings, co‐developing knowledge, solutions, and decision support tools, and implementing the knowledge in the local context–provides effective guidelines to counter the conventional loading dock science.^18^ While this approach is designed to create durable boundary objects that provide foundations for robust conversation and deliberation, we experienced significant barriers to success, including insufficient problem framing, insufficient legitimacy of actors involved, conflicting methodological standards, lack of integration, discontinuous participation, and difficulty tracking scientific and societal impacts. The strategies necessary to overcome these challenges require that researchers and their practitioner partners transcend conventional disciplinary approaches, political practices, and conventional financial agreements and to examine and negotiate basic philosophical and methodological assumptions. For the benefits of transdisciplinary sustainability science to be realized will require nothing short of a reorganization of the knowledge enterprise to more effectively integrate universities and societal stakeholders into complementary systems of knowledge and action, a process envisioned by the OECD in 1972 and which is only partially fulfilled today.

## Conflict of Interest

The authors declare no conflict of interest.
